# O05 Active Bechet's with life threatening arterial disease, complicated by concurrent COVID-19 infection at the peak of the COVID-19 pandemic: did immunosuppression help or hinder?

**DOI:** 10.1093/rap/rkaa053.004

**Published:** 2020-11-03

**Authors:** Rameez Arif, Caroline Cardy

**Affiliations:** Worcestershire Royal Hospital, Worcester, United Kingdom

## Abstract

**Case report - Introduction:**

Behcet’s disease is a rare chronic multi-organ inflammatory disorder characterized by oral aphthous and genital ulceration, often associated with several systemic manifestations. These include skin lesions, arthritis, ocular disease, CNS, GI, and vascular involvement. Arterial disease can affect medium and large sized arteries, leading to aneurysm development with the potential to rupture. We report a case of Behcet’s disease presenting in such a manner, with multiple aneurysms at imminent risk of arterial rupture. The presentation occurred at the peak of the COVID-19 pandemic, in April 2020 with the patient contracting the virus complicating cyclophosphamide immunosuppression therapy post operatively.

**Case report - Case description:**

A 54-year-old man had been under rheumatology follow up for numerous years. He reported recurrent oral ulcers, and episodic fevers. He previously had confirmed cerebral sinus thrombosis, aseptic endocarditis, multiple pulmonary emboli and was investigated for inflammatory bowel disease.

He presented acutely to AE with a painful, tender left calf and a large pulsatile mass in the calf. He reported paraesthesia in his left leg/foot suggestive of nerve compression. CT angiogram performed, revealed a large 6.5cm x 4cm left tibioperoneal trunk (TPT) pseudoaneurysm (Fig.1), and 1.2cm right anterior tibial (ATA) pseudoaneurysm (Fig 2). There was also a 3.8cm infrarenal inflammatory abdominal aortic pseudoaneurysm (Fig 3), and finally a 2.3cm right lower lobe pulmonary artery aneurysm. Laboratory investigations revealed raised inflammatory markers, with ESR of 120 and CRP 73. A diagnosis of vascular Behcet’s was suspected.

Urgent surgical intervention was deemed necessary to prevent limb and life-threatening complications of arterial rupture. He underwent open surgical decompression and graft repair of the left TPT aneurysm. Endovascular stenting of infrarenal AAA was performed and the right ATA aneurysm was ligated and excised. The pulmonary artery aneurysm was managed conservatively, but interval CT Imaging post immunosuppression treatment showed complete resolution. This undoubtedly was due to immunosuppression therapy.

The first cyclophosphamide and methylprednisolone infusion were given day 1 post-op. The following day he developed a fever and a dry cough. He subsequently tested positive for SARS-COV-2. This was at the height of the COVID-19 pandemic. He required supplementary oxygen, but his condition did not deteriorate further, and he made a complete recovery. A difficult dilemma was posed between immunosuppression and the risk of infection having been diagnosed with COVID-19. An MDT approach was adopted, and input was sought from the Birmingham Bechet’s centre of excellence. He continued immunosuppressive treatment once recovered from the virus.

**Case report - Discussion:**

Bechet’s disease is considered a form of systemic vasculitis and is unique in its ability to involve blood vessels of all sizes from the arterial and venous systems. Vascular manifestations in Bechet’s disease are associated with increased mortality and morbidity with manifestations more common in men. Arterial rupture is the leading cause of sudden death in patients with Bechet’s disease. Endovascular and perivascular inflammation of large vessels can lead to haemorrhage, aneurysm formation with risk of rupture, stenosis, and thrombus development.

To prevent potential life-threatening complications of vascular Bechet’s, a combination of surgical/radiological and medical management is often required. High dose corticosteroids and cyclophosphamide infusions are typically used, with aim to achieve disease remission. It is recommended that if surgical/radiological interventions are indicated, it should ideally be performed when the disease is quiescent. However, urgent intervention is required if the aneurysm is at high risk of rupture, rapidly enlarging or if there is organ threatening ischemia. This was the approach used in this patient.

Potent immunosuppression with cyclophosphamide and high dose corticosteroids is indicated in arterial disease of Bechet’s. The inherent risk of infection with these agents, being used at the peak of a global pandemic created a challenging clinical dilemma. To complicate matters further, the patient developed fever and cough following the 1st cyclophosphamide/steroid treatment and tested positive for COVID-19. His condition was relatively mild however and made a full recovery from the virus.

Behcet’s disease can be a difficult and challenging condition to diagnose. There is often a delay in diagnosis. Our patient was previously referred to a tertiary Bechet’s centre but felt there was insufficient clinical grounds to make the diagnosis at that stage.

**Case report - Key learning points:**

This case provides a rare example of an immunosuppressed patient with rheumatic disease contracting COVID-19 after extensive vascular intervention. A poor outcome may have been expected but in contrary the patient’s disease course was mild. This perhaps supports the theory that immunosuppression may play a protective role during COVID-19.

The role of immunosuppression in a hyperinflammatory COVID-19 state is an area of much research interest. Suppression of the cytokine storm with immune therapy has been hypothesised as a potential therapy target for some COVID-19 patients. However, this is still unproven. Corticosteroid treatment with dexamethasone has been shown to reduce mortality in COVID-19 patients in hospital, through the Recovery Trial. In addition, encouraging evidence continues to emerge for the role of IL1 and IL6 inhibitors in the cytokine storm.

It is rare for a patient to present with such extensive aneurysmal disease. Vascular complications of Bechet’s although rare need to be considered.

The management for vascular manifestations of Bechet’s disease requires co-ordination and collaborative work between Rheumatologists, Vascular surgery, and Radiologists. Guidance from expert centres is highly recommended. Prompt medical and surgical intervention is needed to prevent serious limb and life-threatening complications of arterial Bechet’s disease.

Our case raises the clinical conundrum of immunosuppression therapy in serious organ threatening rheumatic disease with concomitant COVID-19 infection. A patient-centred approach with patient involvement in clinical decisions is required. Patients and clinicians alike should balance the risks associated with organ threatening rheumatic disease and infection risks. The current global coronavirus pandemic makes such decisions more difficult with an additional communicable disease prevalent in the community. With few clinically proven therapies and no vaccine available for COVID-19, Rheumatologists will have to grapple with this dilemma for the foreseeable future.

